# Supplementary material for: The causal relationship between the human gut microbiota and pyogenic arthritis: a Mendelian randomization study
Source: Front Cell Infect Microbiol. 2024 Nov 26;14:1452480. doi: 10.3389/fcimb.2024.1452480 (PMC11629706; doi:10.3389/fcimb.2024.1452480)
Supplement: Supplementary file 1 [file DataSheet1.zip › mendelian test/ebi-a-GCST90027704.csv_leave_one_out.pdf]

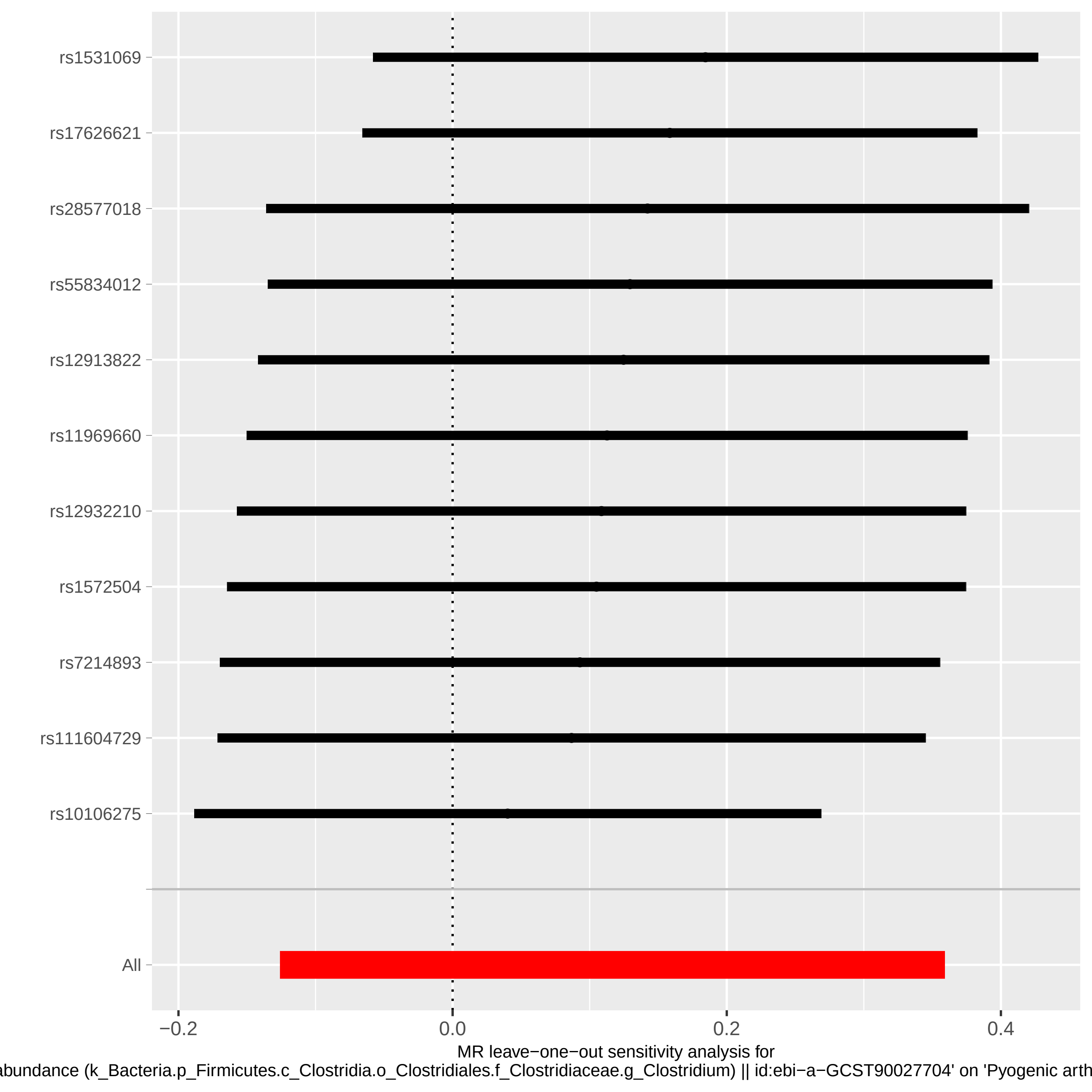

rs1531069
rs17626621
rs28577018
rs55834012
rs12913822
rs11969660
rs12932210
rs1572504
rs7214893
rs111604729
rs10106275
All
−0.2
0.0
0.2
0.4
MR leave−one−out sensitivity analysis for
abundance (k_Bacteria.p_Firmicutes.c_Clostridia.o_Clostridiales.f_Clostridiaceae.g_Clostridium) || id:ebi−a−GCST90027704' on 'Pyogenic arth
